# Human Immunodeficiency Virus-Associated Gastrointestinal Disease: Common Endoscopic Biopsy Diagnoses

**DOI:** 10.4061/2011/247923

**Published:** 2011-04-26

**Authors:** Feriyl Bhaijee, Charu Subramony, Shou-Jiang Tang, Dominique J. Pepper

**Affiliations:** ^1^Department of Pathology, University of Mississippi Medical Center, 2500 North State Street, Jackson, MS 39216, USA; ^2^Department of Medicine, University of Mississippi Medical Center, 2500 North State Street, Jackson, MS 39216, USA

## Abstract

The gastrointestinal (GI) tract is a major site of disease in HIV infection: almost half of HIV-infected patients present with GI symptoms, and almost all patients develop GI complications. GI symptoms such as anorexia, weight loss, dysphagia, odynophagia, abdominal pain, and diarrhea are frequent and usually nonspecific among these patients. Endoscopy is the diagnostic test of choice for most HIV-associated GI diseases, as endoscopic and histopathologic evaluation can render diagnoses in patients with non-specific symptoms. In the past three decades, studies have elucidated a variety of HIV-associated inflammatory, infectious, and neoplastic GI diseases, often with specific predilection for various sites. HIV-associated esophageal disease, for example, commonly includes candidiasis, cytomegalovirus (CMV) and herpes simplex virus (HSV) infection, Kaposi's sarcoma (KS), and idiopathic ulceration. Gastric disease, though less common than esophageal disease, frequently involves CMV, *Mycobacterium avium-intracellulare* (MAI), and neoplasia (KS, lymphoma). Small bowel biopsies and intestinal aspirates from HIV-infected patients often show HIV enteropathy, MAI, protozoa (Giardia, Isospora, Cryptosporidia, *amebae*, Microsporidia), and helminths (*Strongyloides stercoralis*). Colorectal biopsies demonstrate viral (CMV, HSV), bacterial (Clostridia, Salmonella, Shigella, *Campylobacter*), fungal (cryptococcosis, histoplasmosis), and neoplastic (KS, lymphoma) processes. Herein, we review HIV-associated GI pathology, with emphasis on common endoscopic biopsy diagnoses.

## 1. Introduction

In 2004, the World Health Organization (WHO) identified HIV/AIDS as the world's most urgent public health challenge, as AIDS represents the greatest lethal epidemic in recent history. The gastrointestinal (GI) tract is a major site of disease in HIV infection: almost half of all HIV-infected patients present with GI symptoms, and almost all patients develop GI complications [1]. GI symptoms, such as anorexia, weight loss, dysphagia, odynophagia, abdominal pain, and diarrhea, are common and usually non-specific in this population. Endoscopy is the diagnostic test of choice for most HIV-associated GI diseases, as endoscopic and histopathologic evaluation can render diagnoses in patients with non-specific symptoms. 

Pathologic evaluation of endoscopic biopsy specimens includes light and electron microscopy, special stains, immunohistochemical techniques, fluorescent in situ hybridization (FISH), and polymerase chain reaction (PCR) [2]. Light microscopy, with hematoxylin and eosin (H & E) staining, is often sufficient to suggest or confirm a diagnosis. Electron microscopy may be used to identify protozoan infections of the small bowel, as small organisms such as Cryptosporidia and microsporidia are more readily apparent with this technique. Special stains can be used to highlight specific disease characteristics: periodic acid-Schiff with diastase (PASD) stains highlight acid mucopolysaccharides, glycogen, and pseudohyphae in candidiasis; Grocott's methenamine silver (GMS) stains reveal fungal elements such as *Candida,* Histoplasmosis, and *Cryptococcus*; acid-fast bacilli (AFB) stains demonstrate mycobacterial bacilli, as in *mycobacterium avium-intarcellulare* (MAI) infection; Warthin-Starry stains are used for spirochetes; and Brown-Brenn stains aid in the diagnosis of microsporidia. Immunohistochemistry, FISH, and PCR are valuable tools to identify viral infections—such as cytomegalovirus (CMV) or herpes simples virus (HSV)—and HIV-associated neoplasia in tissue samples. 

Herein, we review HIV-associated GI pathology, with emphasis on common endoscopic biopsy diagnoses.

## 2. Materials and Methods

We reviewed the current medical literature. Images were selected from archives in the Departments of Pathology and Gastroenterology at a large academic hospital.

## 3. Results and Discussion

### 3.1. Upper GI Pathology (Tables 1 and 2; Figures 1 and 2)

**Figure 1:**
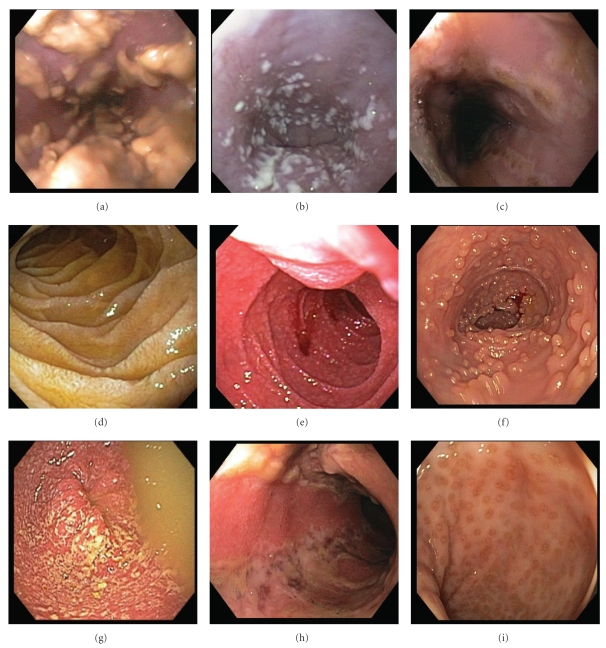
Endoscopic features of HIV-associated GI pathology. (a, b) Esophageal candidiasis: creamy mucosal plaques, (c) esophageal ulceration, (d) duodenal giardiasis, (e) intestinal cryptosporidiosis, with granular, friable mucosa, (f) ileal lymphoid hyperplasia, (g) bacterial colitis, (h) colonic ulceration, and (i) proctitis.

**Figure 2:**
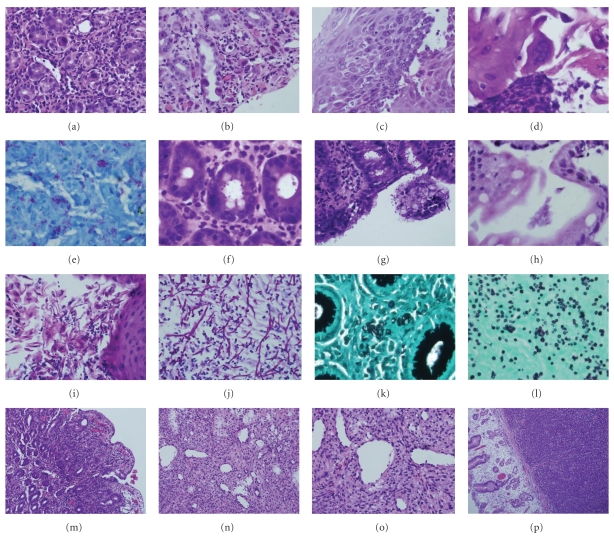
Histopathologic features of HIV-associated GI pathology. (a) CMV gastritis, (b) CMV vasculitis, (c) HSV esophagitis, (d) HSV cytopathic effect, (e) *Mycobacterium avium-intercellulare* (acid fast stain), (f, g) cryptosporidiosis, (h) giardiasis, (i) esophageal candidiasis, (j) candidal pseudohyphae and spores (PASD stain), (k) cryptococcosis (GMS stain), (l) histoplasmosis (GMS stain), (m) HIV enteropathy, (n, o) Kaposi sarcoma, and (p) gastric non-Hodgkin's lymphoma.

Definitive diagnosis of HIV-associated esophageal diseases requires upper endoscopy with biopsy, as the most common esophageal lesions include candidiasis, CMV and HSV infections, and idiopathic ulceration. Endoscopically, candidiasis appears as white or yellow plaques with surrounding mucosal erythema, on otherwise intact esophageal mucosa (Figure 1). The plaques are usually discrete, but may become confluent and/or circumferential. Esophageal ulceration suggests concomitant viral infection (such as CMV or HSV) [3]. Histopathologic features of candidiasis include pseudomembranes and neutrophilic infiltrates; the fungi appear as budding yeast forms and pseudohyphae (Figure 2). 

CMV is the most common opportunistic agent in HIV-infected patients, and, while it can affect the entire GI tract, it frequently involves the esophagus and the colon [4]. CMV typically presents with distal esophageal ulceration, which may range from small, discrete, and superficial to extremely large and deep, such that only infected granulation tissue is evident (Figure 1). Rarely, CMV may present as a mass lesion, or inflammatory pseudotumor, causing luminal obstruction. CMV-infected cells are enlarged (10–15 *μ*m), with strongly eosinophilic nuclear inclusions, which are surrounded by a clear halo, and small basophilic cytoplasmic inclusions; these cytoplasmic inclusions cause a granular appearance under light microscopy (Figure 2) [2]. Infected and dead cells may appear mummified and enlarged, but without nuclear inclusions. Biopsies from the ulcer base are more likely to demonstrate characteristic CMV inclusions, which are usually seen in stromal and endothelial cells, as compared to HSV infection, which manifests in squamous cells [3]. Necrotizing vasculitis and regenerative mucosal hyperplasia may also be present (Figure 2) [2]. Immunohistochemistry, FISH, and PCR are ancillary to microscopic diagnosis and offer greater sensitivity in latent infection.

Endoscopic features of HSV infection include vesiculation and ulceration; the latter are round, multiple, well circumscribed, uniform, and smaller than those in CMV disease (Figure 1). Histopathologic features of HSV are more evident in biopsies from ulcer edges, as opposed to ulcer bases, and include necrosis, acute and chronic inflammation, and diagnostic nuclear changes (Figure 2). HSV cytopathic effect encompasses (i) homogenous ground-glass nuclei with margination of chromatin, (ii) nuclear molding, (iii) multinucleation, and, rarely, (iv) Cowdry type A nuclear inclusions [5]. Moreover, HSV-induced ulceration exhibits an inflammatory infiltrate rich in neutrophils and histiocytes, the presence of which should prompt a thorough search for viral cytopathic changes [2].

HIV-associated idiopathic esophageal ulceration refers to large, irregular, mid esophageal and distal esophageal ulcers with no identifiable etiologic agent. These ulcers are typically associated with severe odynophagia and weight loss, but often respond to corticosteroid and/or thalidomide therapy [3]. Endoscopically, idiopathic ulcers resemble CMV-induced ulcers, but are more often solitary and deep [6]. Biopsy reveals a nonspecific mixed inflammatory infiltrate, with prominent eosinophils and granulation tissue [5]. Other esophageal diseases in HIV-infected patients include gastroesophageal reflux disease (GERD) with esophagitis or peptic strictures, pill-induced esophagitis, carcinoma, tuberculosis, histoplasmosis, cryptosporidiosis, Kaposi's sarcoma, and Pneumocystis infection [3]. 

Many opportunistic GI diseases have decreased in frequency since the introduction of effective antiretroviral therapy, but *Helicobacter pylori* infection and GERD have apparently increased in frequency [7]. This may be attributed, in part, to alleviation of the gastric hypochloridia often seen in severely immunocompromised patients.

While upper GI symptoms, such as epigastric pain, nausea, and vomiting, are often reported by HIV-infected patients, gastric disease is relatively uncommon [3]. In HIV-infected patients, CMV is the most frequent opportunistic gastric infection and may be the most commonly identified cause of ulcer disease in symptomatic patients [8, 9]. Endoscopically, CMV gastritis appears as patchy erythema, erosions, or multiple small ulcers. Unlike intestinal biopsy specimens, gastric biopsies often show CMV inclusions in epithelial cells. CMV-infected cells are markedly enlarged with amphophilic nuclear inclusions and abundant granular basophilic cytoplasm (Figure 2) [5]. 

*Mycobacterium-avium intracellulare* (MAI) is the most common mycobacterial infection in severely immunocompromised patients, and disseminated infection often causes mesenteric lymphadenitis with diffuse abdominal pain, weight loss, fever, or diarrhea. Endoscopically, MAI commonly presents with multiple raised nodules or normal-appearing mucosa. Less common findings include ulceration, erythema, edema, friability, reduced mucosal vascularity, stricture, and aphthous erosions [10]. Definitive diagnosis is established by biopsy, with demonstration of acid-fast bacilli in tissue specimens; blood and stool cultures may also reveal MAI infection [3]. Antimycobacterial therapy for MAI is prolonged and can be complicated by adverse drug effects.

In addition to opportunistic infections, gastric neoplasia may be diagnosed on biopsies: Kaposi's sarcoma (KS) and non-Hodgkin's lymphoma frequently affect the stomach. Gastrointestinal Kaposi's sarcoma is often asymptomatic, but gastric involvement may result in pyloric obstruction, pain, or upper GIT bleeding [3]. Endoscopically, KS appears as submucosal violet-red nodules, plaques, or polyps. Its subtle, submucosal presentation often results in false-negative endoscopic biopsy results. Histologically, KS is composed of spindle cells and irregular, jagged, slit-like spaces lined by atypical endothelial cells; extravasated red cells, hemosiderin, and eosinophilic round cytoplasmic globules are also often evident (Figure 2) [5]. Moreover, human herpesvirus 8 (HHV8) immunostains may aid in diagnosis, as the virus has been implicated in the pathogenesis of KS. In contrast to KS, gastric lymphoma is frequently symptomatic, as it is typically advanced (stage IV) at presentation, and causes epigastric pain or symptoms of gastric outlet obstruction [3]. The majority (95%) are B-cell lymphomas—usually immunoblastic or large cell type [11] (Figure 2)—and can be difficult to distinguish from adenocarcinoma on endoscopy, because both entities present as ulcerated mass lesions. Biopsy, however, facilitates definitive diagnosis.

### 3.2. HIV-Associated Diarrhea

Diarrhea is the most common GI symptom in HIV-infected patients, affecting up to 90% of patients, and increases in frequency and severity as immune function deteriorates [12]. The most common HIV-associated diarrheal pathogens include *Cryptosporidium*, nontubercular mycobacteria, microsporidia, bacteria (*Salmonella, Shigella, Campylobacter, and E. coli*), and CMV [13].

AIDS-related diarrhea results from numerous etiologic agents and pathophysiologic mechanisms [14]. HIV enteropathy, for example, causes ileal dysfunction, altered bowel motility, and bacterial overgrowth. Direct HIV infection of intestinal mucosa results in chronic inflammation, infection of enterochromaffin cells, gp120 interation with VIP receptors, antiproliferative effects, and enterocyte dysfunction. MAI leads to an exudative enteropathy and altered secretion of inflammatory mediators (e.g., interleukin-1). *C. difficile* also alters secretion of IL-1. *Cryptosporidium,* microsporidia, and *Isospora* cause diarrhea by decreasing the mucosal surface area.

Despite the controversial role of bidirectional endoscopy in the evaluation of HIV-associated diarrhea, the following diagnostic algorithm has been suggested: in patients with diarrhea, a CD4 count less than 100/mm^3^ and inconclusive routine stool studies, the best overall diagnostic test is colonoscopy with terminal ileal intubation and biopsy; in patients with CD4 counts of 100–200/mm^3^, flexible sigmoidoscopy and biopsy are sufficient, because CMV infection is less likely to occur at higher CD4 counts [3]. If diagnostic doubt remains, duodenal biopsies may be useful, especially in patients with lower CD4 counts [13]. The role of routine stool studies for ova, parasites, and so forth—*before* endoscopy and biopsy—cannot be overemphasized.

### 3.3. HIV-Associated Small Bowel Pathology (Tables 1 and 2; Figures 1 and 2)

Intestinal biopsies and aspirates from HIV-infected patients may reveal HIV enteropathy, CMV, MAI, protozoa (*Cryptosporidium*, *Isospora, microsporidia, Giardia*), helminths (*Strongyloides stercoralis*), and fungi (*Histoplasma capsulatum*)—all of which may cause malabsorption and/or diarrhea. The organisms most commonly found on duodenal biopsies include CMV, *Cryptosporidium*, microsporidia, and Giardia [3]. 

As in gastric infection (see above), CMV causes intestinal inflammation, erosion, and ulceration, with characteristic CMV inclusions in stromal and endothelial cells. Intestinal MAI causes subtle macroscopic changes, such as thickened or edematous mucosal folds with occasional yellow patches, which can become confluent [3]. Histopathologic features of intestinal MAI infection mimic Whipple's disease, as both entities exhibit periodic acid-Schiff-(PAS)-positive foamy macrophages in the lamina propria: in MAI, however, the macrophages are filled with acid-fast bacilli and villous atrophy is often present (Figure 2) [5].

Small bowel protozoal infections cause irregular, fused, widely-spaced, shortened villi, resulting in an erythematous, granular appearance on endoscopy (Figure 1) [5]. *Cryptosporidia* are small (2–5 *μ*m), round, basophilic organisms, which appear on the luminal borders of enterocytes, submucosal glands, and ducts on light microscopy (Figure 2); surrounding villi are variably atrophic, with infrequent crypt abscesses and neutrophils in the lamina propria. Both Cryptosporidia and microsporidia are often missed on routine biopsies, as their subtle appearance is better appreciated with electron microscopy. If microsporidia spores are not identified in stool samples or duodenal aspirates, Brown-Brenn stains may aid in visualization. Moreover, microsporidia can be reliably diagnosed using a modified trichrome stain, PCR analysis of stool specimens, or FISH. Enteric isosporiasis, secondary to opportunistic *Isospora belli* infection, is more common in HIV-infected patients in developing countries than in the United States or in Europe. Isosporiasis localizes to the small bowel, but can spread to the colon and regional lymph nodes. Diagnosis is facilitated by identification of (i) oocysts in stool samples or duodenal aspirates, or (ii) merozoites on light or electron microscopy. 

*Giardia lamblia* is a flagellated enteric protozoan, which is typically waterborne, but is also transmitted person-to-person through fecal-oral spread [2, 15]. Giardia causes acute and chronic diarrhea worldwide; in the USA, it is the most commonly identified intestinal parasite. Acute giardiasis causes diarrhea, cramps, bloating, and flatulence; chronic infection results in malabsorption, steatorrhea, malaise, weight loss, and diffuse abdominal discomfort. Initially transmitted as a cyst, Giardia forms a trophozoite that adheres to intestinal epithelium. The trophozoites are pearshaped and binucleate, but may appear sickle-shaped in sagittal sections, and mistaken for detached enterocytes (Figure 2). Identification of cysts or trophozoites in the stool confirms the diagnosis of giardiasis. In the absence of cysts of trophozoites in stool specimens, upper endoscopy and duodenal biopsy may confirm the diagnosis. Giardia's pathogenicity is influenced by cellular and humoral immune responses, and certain immunodeficiencies—such as HIV infection—predispose to giardiasis. HIV-infected patients show a decreased antibody response to Giardia, but their clinical manifestations are similar to those of immunocompetent hosts [15]. Metronidazole is effective in the treatment of giardiasis, but is not recommended for pregnant patients due to potential teratogenicity. 

*Strongyloides stercoralis* is a parasitic nematode with a predilection for the small intestine [2]. Intestinal lesions can be divided into three morphologic categories: (i) catarrhal enteritis, in which the small bowel appears congested with abundant mucus secretion, petechial hemorrhages, and submucosal mononuclear inflammation; (ii) edematous enteritis, in which the bowel wall is thickened, the mucosal folds are flattened, and the submucosa shows edema and inflammation; (iii) ulcerative enteritis, in which the bowel wall is rigid, fibrotic, and ulcerated, with abundant neutrophilic infiltrates.

HIV enteropathy often causes severe malabsorption, lactose intolerance, vitamin B12 and D-xylose malabsorption, and increased intestinal permeability with protein loss [3]. While the pathophysiology of HIV enteropathy-induced diarrhea remains unclear, there are currently two hypothetical mechanisms: (i) mucosal HIV infection affects intestinal permeability by disrupting tight junctions, epithelial apoptotic activity, or both [16]; (ii) coupled with a coreceptor, HIV protein gp120 causes calcium-mediated microtubule loss and cellular instability results [17]. Endoscopically, HIV enteropathy may manifest as “frosted” mucosa [3]; histopathologic changes include villous blunting and widening, vacuolated enterocytes, and increased inflammatory cells in the lamina propria. Initiation of highly active antiretroviral therapy (HAART) leads to resolution of both clinical symptoms and ultrastructural tissue changes.

### 3.4. Lower GI Pathology (Tables 1 and 2; Figures 1 and 2)

The endoscopic appearance of viral colitis in HIV-infected patients is highly variable [18]. Evaluation of the colon may reveal (i) normal mucosa, (ii) characteristic features of viral infection (such as nuclear inclusions involving surface epithelium), or (iii) severe ulceration (such as in CMV colitis). CMV is the most common viral cause of diarrhea in HIV infection and may affect any portion of the GIT. In HIV-infected patients, latent CMV reactivates, leading to viremia, deposition of viral particles in the vascular endothelium, and vasculitis; this culminates in submucosal ischemia and ulceration [19]. To identify CMV colitis, colonoscopy is preferred over flexible sigmoidoscopy, as more than one third of affected patients have gross disease restricted to proximal regions of the colon [20]. Endoscopically, CMV colitis may appear as (i) colitis alone (with edema and subepithelial hemorrhage), (ii) colitis with ulceration, or (iii) aggregates of discrete ulcers (ranging from 5 mm to 2 cm) surrounded by unremarkable mucosa (Figure 1) [21, 22]. Occasionally, the colonic mucosa may appear granular and friable, as in ulcerative colitis, or the patient may present with an abdominal mass [18]. 

CMV colitis is diagnosed by identification of typical inclusion bodies in biopsy specimens (see above) [18]. Serologic studies and cultures of biopsy materials are unnecessary. Viral inclusions are most often present in the endothelial cells of the deeper layers of the bowel wall, thus multiple biopsies may be necessary for identification. Necrotizing vasculitis and endoluminal thrombosis may also be seen. As in CMV infection elsewhere in the GI, the etiologic role of CMV in the cellular inclusions can be confirmed by immunoperoxidase staining, FISH, or PCR. Symptomatic treatment of CMV colitis includes intravenous ganciclovir, foscarnet, or cidofovir, but none of these agents improve mortality [23].

Bacterial colitis can result from (i) anomalous growth of intestinal flora, (ii) bacterial infections (including Salmonella, Shigella, and *Campylobacter*), or (iii) opportunistic pathogens (such as atypical mycobacteria) [2]. Endoscopically, bacterial colitis appears as epithelial necrosis, abscesses, erosions, ulceration, and focal hemorrhage. Histopathologic features of bacterial colitis include mucosal epithelial changes: degeneration, necrosis, cellular atypia, regeneration, and fissures. The severity of immunodeficiency and bacterial infection determines the degree of mucosal damage. On H&E staining, bacterial forms appear as small, basophilic cocci, bacilli, or coccobacilli. MAI may also affect the colon, causing diarrhea, malabsorption, flattened mucosa on endoscopic examination, and a dense histiocytic infiltrate in the lamina propria. In this setting, Crohn's disease may be considered in the differential diagnosis. As in upper GI MAI infection, acid-fast staining will reveal mycobacterial organisms.

HIV-related cryptococcosis commonly affects the colon and the esophagus, while the stomach and terminal ileum are rarely involved [24]. Endoscopically, *Cryptococcus* infection causes destructive cysts composed of fungal masses. Histopathologic features include fungal colonies of spherical to oval yeast-like pleomorphic cells, which are often surrounded by a clear halo corresponding to the mucinous capsule (Figure 2). Mucicarmine, Alcian blue, and GMS stains facilitate identification of fungal forms.

Histoplasmosis is the most common endemic mycosis in HIV-infected patients; this population is also more likely to experience disseminated infection [25]. Despite frequent GI involvement, the symptoms are typically non-specific or absent [26, 27]. Previous studies of HIV-associated GI histoplasmosis report fever and abdominal pain in 70% of patients, and weight loss and diarrhea in less than 50% [26, 27]. GI bleeding, obstruction, perforation, and strictures are rare. In HIV-infected patients, histoplasmosis is most likely to affect the ileocecal region, with marked morphologic heterogeneity [28]. The following pathologic features may be seen: (i) grossly unremarkable mucosa, despite marked histiocytic infiltration of the lamina propria, (ii) infected macrophages causing plaques and pseudopolyps, and (iii) ulceration with tissue necrosis. GMS staining often highlights the fungal elements (Figure 2). 

In HIV infection and men who have sex with men, proctitis may occur secondary to HSV, *Neisseria gonorrhea, Chlamydia trachomatis,* or *Treponema pallidum* infection [29]. HSV proctitis presents with anorectal burning, pruritis, tenesmus, diarrhea, constipation, mucoid or bloody stools, and/or bilateral tender inguinal lymphadenopathy [30]. Approximately 1–3 weeks after initial infection, multiple vesicles erupt on the perineum and in the anal canal and rectum; these vesicles evolve into aphthous ulcers (Figure 1) [18]. Endoscopically, small single or grouped vesicles surrounded by erythema are seen in the distal 5–10 cm of the rectum; these vesicles eventually coalesce to form aphthous ulcers. Rectal biopsies may reveal typical intranuclear inclusion bodies or multinucleated giant cells. As in CMV infection, viral culture is rarely necessary. Symptomatic treatment includes sitz baths, topical anesthetics, oral analgesia, and antiviral agents (such as acyclovir, valacyclovir, and famciclovir) [30].

### 3.5. HIV-Associated GI Bleeding

With increasing immunosuppression, HIV-infected patients with GI bleeding may have unique lesions related to HIV disease and should be evaluated endoscopically [3]. Kaposi's sarcoma and gastroduodenal lymphoma are the most common causes of HIV-related upper GI bleeding in advanced HIV infection. Upper endoscopy with biopsy facilitates diagnosis of both entities, and endoscopic therapy may temporarily control bleeding, but the definitive therapy is often surgical resection [3]. Lower GI bleeding in advanced HIV infection may result from CMV colitis or Kaposi's sarcoma [3]. Anorectal bleeding may be secondary to condylomata, HSV, idiopathic ulceration, and non-Hodgkin's lymphoma [3].

## 4. Conclusions

Despite widespread use of HAART in HIV infection, the GI tract is still frequently affected by HIV-associated disease processes. HIV-infected patients often present with non-specific GI symptoms, and the diagnostic workup requires consideration of both individual risk factors and common HIV-associated GI diseases. A symptom-based approach to diagnosis will direct clinical evaluation, with the caveat that Occam's razor—that is, diagnostic parsimony—frequently does not apply in HIV-infected patients [31]. Therefore, identification of one pathologic entity should not forestall the search for concomitant disease processes. Endoscopy and biopsy, with histopathologic and microbiologic analysis, are frequently required to identify and treat GIT disease in HIV-infected patients.

Numerous studies over the past three decades have elucidated a variety of HIV-associated inflammatory, infectious, and neoplastic diseases of the GI tract, often with specific predilection for various sites. HIV-associated esophageal disease, for example, commonly includes candidiasis, CMV and HSV infection, Kaposi's sarcoma, and idiopathic ulceration. Gastric disease, though less common than esophageal disease, frequently involves CMV, *Mycobacterium avium intracellulare* (MAI), and neoplasia (KS, lymphoma). Small bowel biopsies and intestinal aspirates from HIV-infected patients often show HIV enteropathy, MAI, protozoa (Giardia, Isospora, Cryptosporidia, *amebae*, Microsporidia), and helminths (*Strongyloides stercoralis*). Colorectal biopsies demonstrate viral (CMV, HSV), bacterial (Clostridia, Salmonella, Shigella, *Campylobacter*), fungal (cryptococcosis, histoplasmosis), and neoplastic (KS, lymphoma) processes.

In future, the widespread use of effective antiretroviral therapy will likely decrease the frequency and severity of HIV-related illnesses, and advances in medical knowledge and technology will improve the prevention, diagnosis, and treatment of HIV-associated GI disease.

## Figures and Tables

**Table 1:** HIV-associated GI pathology: common biopsy diagnoses.

Pathologic process	Esophagus	Stomach	Small bowel	Colorectal
Inflammatory/Ulcerative	Idiopathic Pill-induced	Nonspecific gastritis	HIV enteropathy	HIV enteropathy Idiopathic

Viral infection	CMV	CMV	CMV	CMV
	HSV			HSV

		MAI	MAI	*Clostridium difficile*
				Salmonella
Bacterial infection	—			Shigella
				*Campylobacter*
				Spirochetosis

			*Giardia lamblia*	
			Cryptosporidia	
Protozoan and Helminthic infections	—	Cryptosporidia	Isospora belli	
			Microsporidia	
			*Strongyloides stercoralis*	

	*Candida albicans*	*Cryptococcus neoformans*	*Histoplasma capsulatum*	Cryptococcus neoformans
Fungal infection	*Candida krusei*			
	*Candida tropicalis*			Histoplasma capsulatum
	*Torulopsis glabrata*			

Neoplastic	Kaposi's sarcoma	Kaposi's sarcoma NHL	Kaposi's sarcoma	Kaposi's sarcoma NHL

Key: HIV: human immunodeficiency virus, CMV = cytomegalovirus, HSV = herpes simplex virus, MAI = *mycobacterium avium-intracellulare*, NHL = non-Hodgkin lymphoma.

**Table 2:** Endoscopic and histopathologic features of common HIV-associated GI diseases [2].

	Viral infections	Bacterial infections	Protozoal and Helminthic infections	Fungal infections	Neoplastic processes
Endoscopy (Figure 1)	Erythema, vesiculation, erosion, ulceration	Hyperemia, ulceration, inflammatory pseudopolyps	Normal to atrophic mucosa	Creamy mucosal patches, ulceration, mycotic abscesses	Mucosal and submucosal mass lesions

Histopathology (Figure 2)	Viral cytopathic effects, inflammatory infiltrates, necrotizing vasculitis	Mucosal epithelial degeneration, loss of intestinal microvilli, cytologic atypia, epithelial fissures, inflammatory infiltrates, abundant foamy macrophages (MAI)	Mucosal atrophy, inflammatory infiltrates, eosinophilia (Isospora), histiocytosis in lamina propria (*Leishmania*)	Cell necrosis, pseudomembranes, hyphae penetrating intact mucosa and vasculature	KS: spindle cell neoplasms with irregular slit-like spaces; NHL: dense proliferation of uniform, small, round, cells
